# Constitution of the Medical Society of South Western New York

**Published:** 1857-07

**Authors:** 


*Constitution of the Medical Society of South Western New York.*—Under this name, a portion—we wish it were larger—of the physicians of Chautauque county, have organized a society, which has now for a year had active existence, designed to afford opportunities for professional improvement, and to advance the interests of physicians generally. It has within its ranks some energetic and earnest men, capable of giving interest and character to its proceedings. The effect of such societies is always to promote harmony and professional *esprit du corps*, and we trust that this one has a prosperous career before it. We notice that the members have established a fee-bill. For ourselves we are inclined to regard its prices as too low, but the great object of a fee-bill is to have some standard of minimum charge, and this will accomplish that end. The society meets four times yearly, at Jamestown and Westfield alternately. We hear that the meetings are well attended and profitable.

